# Quality of Clinical Practice Guidelines for Glycemic Control in Type 2 Diabetes Mellitus

**DOI:** 10.1371/journal.pone.0058625

**Published:** 2013-04-05

**Authors:** Haley K. Holmer, Lauren A. Ogden, Brittany U. Burda, Susan L. Norris

**Affiliations:** 1 Department of Medical Informatics and Clinical Epidemiology, Oregon Health & Science University, Portland, Oregon, United States of America; 2 Kaiser Permanente Center for Health Research, Portland, Oregon, United States of America; University of Colorado Denver, United States of America

## Abstract

**Background:**

Several studies have reported that clinical practice guidelines (CPGs) in a variety of clinical areas are of modest or variable quality. The objective of this study was to evaluate the quality of an international cohort of CPGs that provide recommendations on pharmaceutical management of glycemic control in patients with type 2 diabetes mellitus (DM2).

**Methods and Findings:**

We searched the National Guideline Clearinghouse (NGC) on February 15th and June 4th, 2012 for CPGs meeting inclusion criteria. Two independent assessors rated the quality of each CPG using the Appraisal of Guidelines for Research & Evaluation II (AGREE II) instrument. Twenty-four guidelines were evaluated, and most had high scores for clarity and presentation. However, scope and purpose, stakeholder involvement, rigor of development, and applicability domains varied considerably. The majority of guidelines scored low on editorial independence, and only seven CPGs were based on an underlying systematic review of the evidence.

**Conclusions:**

The overall quality of CPGs for glycemic control in DM2 is moderate, but there is substantial variability among quality domains within and across guidelines. Guideline users need to be aware of this variability and carefully appraise and select the guidelines that they apply to patient care.

## Introduction

High quality clinical practice guidelines (CPGs) provide recommendations based on a systematic review of the evidence, an assessment of balance of benefits and harms, and a transparent process for translating evidence to recommendations [1]. CPGs have the potential to influence the care delivered by a large number of healthcare providers and thus the outcomes of patients [2]. The quality of CPGs is therefore critically important. High-quality, or trustworthy guidelines promote the use of effective clinical services, decrease undesirable practice variation, reduce the use of services that are of minimal or questionable value, increase the use of effective but underused services, and target services to populations most likely to benefit [3].

The global burden of diabetes is enormous. Of the estimated 346 million people worldwide with diabetes, 90% have type 2 diabetes mellitus (DM2) [4]. An estimated 3.4 million persons died in 2004 from causes related to elevated blood glucose and the World Health Organization predicts that diabetes-related deaths will double between 2008 and 2030 [4]. Persons with diabetes have at least two times the risk of death than persons without diabetes [4], and morbidity from both macro-and microvascular disease is substantial. There are numerous pharmaceutical classes and specific agents used to treat hyperglycemia in DM2, with different mechanisms, pharmacokinetics, mean effects on blood glucose, and adverse effects.

A number of studies have reported that CPGs in a variety of clinical areas are of modest or variable quality [5], [6], [7], [8], [9]. The objective of this study was to examine the quality of CPGs that include recommendations on pharmacotherapy for glycemic control in DM2.

## Methods

We searched the National Guideline Clearinghouse (NGC) (www.guideline.gov) on February 15^th^ and June 4^th^, 2012 for all guidelines that provided recommendations on pharmacotherapy for glycemic control in persons with DM2. We searched for CPGs on two separate dates because guidelines are continually being revised, updated, or archived in the NGC and we wanted to ensure that we identified all guidelines relevant to our topic and that we did not exclude a guideline because it was archived during the time of our review process.

The NGC is a publicly available online resource for evidence-based CPGs, funded by the United States government and produced by the Agency for Healthcare and Research Quality (AHRQ). For CPGs to be included in the NGC, guidelines must meet the following criteria: 1) the clinical practice guideline contains systematically developed statements that include recommendations, strategies, or information that assists physicians and/or other health care practitioners and patients to make decisions about appropriate health care for specific clinical circumstances; 2) the clinical practice guideline was produced under the auspices of medical specialty associations; relevant professional societies, public or private organizations, government agencies at the Federal, State, or local level; or health care organizations or plans; 3) corroborating documentation can be produced and verified that a systematic literature search and review of existing scientific evidence published in peer reviewed journals was performed during the guideline development; 4) the full text guideline is available upon request in the English language; 5) the guideline was developed, reviewed, or revised within the last 5 years [10].

In addition to meeting the NGC inclusion criteria, our study required that CPGs provided recommendations for glycemic control in any population with DM2, including adults, children, pregnant women, and persons with DM2 and any comorbid condition. If the full guideline was not available in the public domain, we purchased a copy.

Two coauthors with experience in quality assessment of CPGs independently scored each guideline using the Appraisal of Guidelines for Research & Evaluation II (AGREE II) instrument [11] (Table 1). AGREE II consists of 23 items grouped into six domains: 1) scope and purpose; 2) stakeholder involvement; 3) rigor of development; 4) clarity of presentation; 5) applicability; and 6) editorial independence [11]. The assessors then compared their individual scores for each item and came to consensus on discrepant scores (defined as scores varying by three points or more on the seven-point AGREE II scale). This approach accounted for frank error on the part of an assessor, when they had missed the relevant part of the guideline in their original assessment. If the two assessors were unable to reach consensus, a third person was consulted. If the two assessors' scores differed by two points they were averaged; if they differed by one point the lower score was kept. Standardized domain scores (expressed on a scale of 0–100) were calculated using the approach of AGREE II ([obtained score – minimum possible score] divided by [maximum possible score – minimum possible score]) [11]. The overall AGREE II evaluation of recommend, recommend with modifications, or do not recommend each guideline was independently determined by each assessor and then consensus was achieved.

**Table 1:** AGREE II Instrument for the Quality Assessment of Clinical Practice Guidelines.

AGREE II Domain	AGREE II Item
Scope and Purpose	The overall objective(s) of the guideline is (are) specifically described.
	The health question(s) covered by the guideline is (are) specifically described.
	The population (patients, public, etc.) to whom the guideline is meant to apply is specifically described.
Stakeholder Involvement	The guideline development group includes individuals from all relevant professional groups.
	The views and preferences of the target population (patients, public, etc.) have been sought.
	The target users of the guideline are clearly defined.
Rigor of Development	Systematic methods were used to search for evidence.
	The criteria for selecting the evidence are clearly described.
	The strengths and limitations of the body of evidence are clearly described.
	The methods used for formulating the recommendations are clearly described.
	The health benefits, side effects, and risks have been considered in formulating the recommendations.
	There is an explicit link between the recommendations and the supporting evidence.
	The guideline has been externally reviewed by experts prior to its publication.
	A procedure for updating the guideline is provided.
Clarity and Presentation	The recommendations are specific and unambiguous.
	The different options for management of the condition or health issue are clearly presented.
	Key recommendations are easily identifiable.
Applicability	The guideline describes facilitators and barriers to its application.
	The guideline provides advice and/or tools on how the recommendations can be put into practice.
	The potential resource implications of applying the recommendations have been considered.
	The guideline presents monitoring and/or auditing criteria.
Editorial Independence	The views of the funding body have not influenced the content of the guideline.
	Competing interests of guideline development group members have been recorded and addressed.

AGREE II, Appraisal of Guidelines for Research and Evaluation II [11].

CPGs were considered to be based on a systematic review if there was either reference to a review or a review was contained within the guideline document, the review reported a search of one or more bibliographic databases, and a defined cohort of studies derived from the search was used to formulate recommendations.

## Results

Twenty-four guidelines met our inclusion criteria (Table 2; Figure S1). Ten of the guidelines were published between 2007 and 2009; the remainder were published in 2010 or later. The majority of the CPGs (n = 14; 58%) were developed by US-based organizations, followed by European (n = 5; 21%), Canadian (n = 3; 13%), and international (n = 2; 8%) organizations. The CPGs meeting inclusion criteria were developed primarily by non-profit organizations (25%), government agencies (21%), and medical specialty societies (21%).

**Table 2:** Quality Assessment of Guidelines for Glycemic Control in Type 2 Diabetes Mellitus.

	AGREE II Domain						
Clinical Practice Guideline	Scope and purpose	Stakeholder involvement	Rigor of development	Clarity and presentation	Applicability	Editorial independence	Overall assessment
AACE [35]	83%	39%	48%	83%	29%	33%	Recommend with modifications
ACP I [22]	94%	44%	79%	67%	33%	67%	Recommend
ACP II [23]	94%	44%	79%	94%	29%	75%	Recommend
ADA [36]	56%	50%	38%	67%	58%	33%	Recommend with modifications
AMDA [37]	44%	50%	17%	94%	38%	0%	Recommend with modifications
CADTH I [12]	78%	78%	58%	94%	42%	33%	Recommend
CADTH II [13]	61%	33%	54%	94%	21%	33%	Recommend
CADTH III [38]	67%	89%	56%	78%	33%	42%	Recommend
ESC [39]	67%	44%	50%	61%	29%	8%	Recommend with modifications
ICSI [19]	72%	44%	60%	78%	83%	42%	Recommend
IDC [40]	22%	22%	6%	72%	46%	0%	Would not recommend
IDF [41]	83%	44%	63%	89%	38%	42%	Recommend
JDC [42]	61%	50%	10%	72%	38%	0%	Would not recommend
KDOQI [43]	83%	56%	75%	89%	33%	33%	Recommend
NCC-ACC [14]	83%	72%	81%	78%	63%	8%	Recommend
NCC-WCH [15]	94%	94%	79%	89%	54%	42%	Recommend
NHCHC [21]	44%	44%	4%	72%	33%	8%	Would not recommend
NICE [44]	6%	28%	56%	72%	54%	8%	Recommend with modifications
NY DoH [20]	17%	6%	0%	78%	8%	0%	Would not recommend
QPHC [16]	83%	61%	31%	83%	79%	42%	Recommend
SIGN [17]	94%	94%	81%	83%	83%	25%	Recommend
UMHS [45]	56%	28%	33%	83%	21%	33%	Recommend with modifications
VA/DoD [18]	61%	89%	73%	83%	71%	0%	Recommend
WDPCP [46]	56%	50%	17%	83%	46%	8%	Recommend with modifications
Mean*; Range	64%; 6%–94%	52%; 6%–94%	48%; 0%–81%	81%; 61%–94%	43%; 21%–83%	26%; 0%–75%	

Data presented are AGREE II scores [11]. Each item was rated on a seven-point Likert scale that measured the extent to which an item was fulfilled: 1-strongly disagree to 7-strongly agree. Scores were standardized within domains by dividing the difference between the consensus score and the minimum possible score by the difference between the maximum and minimum possible scores.

(*) Domain scores were averaged across guidelines.

**Figure 1:**
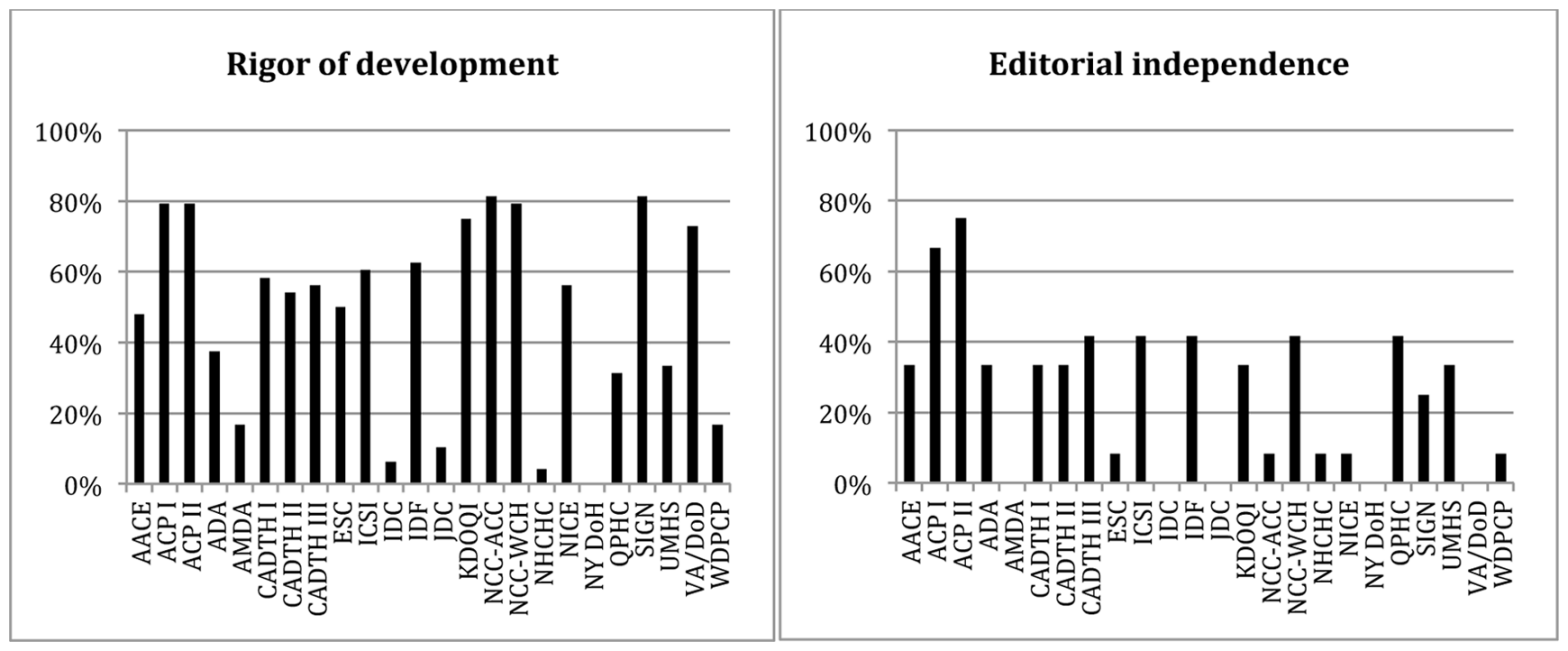
Standardized domain scores for rigor of development and editorial independence. Scores are obtained from two of the domains of AGREE II (Appraisal of Guidelines for Research and Evaluation) [11] Guidelines: American Association of Clinical Endocrinologists (AACE), American College of Physicians (ACP), American Diabetes Association (ADA), American Medical Directors Association (AMDA), Canadian Agency for Drugs and Technologies in Health (CADTH), European Society of Cardiology (ESC), Institute for Clinical Systems Improvement (ICSI), International Diabetes Center (IDC), International Diabetes Federation (IDF), Joslin Diabetes Center (JDC), National Kidney Foundation (KDOQI), National Collaborating Centre for Acute and Chronic Conditions (NCC-ACC), National Collaborating Centre for Women's and Children's Health (NCC-WCH), National Health Care for the Homeless Council (NHCHC), National Institute for Health and Clinical Excellence (NICE), New York State Department of Health (NY DoH), Qatif Primary Health Care (QPHC), Scottish Intercollegiate Guidelines Network (SIGN), University of Michigan Health System (UMHS), Department of Veterans Affairs/Department of Defense (VA/DoD), Wisconsin Diabetes Prevention and Control Program (WDPCP).

Guidelines: See Figure 1 for the list of abbreviations.

The overall quality of the included CPGs varied considerably, both within and across AGREE II domains (Table 2). No guideline scored more than 50% in all six AGREE II domains. Across the CPGs, scores were highest for the domain of clarity and presentation (mean 81% of the maximum possible score). Most of the guidelines presented easily identifiable, specific key recommendations and different options for management of DM2. The domain of scope and purpose was also rated relatively high (mean 64% of the maximum possible score). The overall objectives of the guidelines and the specific populations to whom the guidelines were meant to apply were also well described in most CPGs.

Scores for the stakeholder involvement (mean 52% of the maximum possible score) and applicability (mean 43% of the maximum possible score) were variable across guidelines. Seven guidelines scored greater than 60% on stakeholder involvement [12], [13], [14], [15], [16], [17], [18], while only four CPGs scored that well on applicability [16], [17], [18], [19].

Scores for rigor of development were generally between 30–60%, with a few CPGs scoring very high and a few scoring very low (Figure 1). The Scottish Intercollegiate Guideline Network (SIGN) [17] and National Collaborating Centre for Women's and Children's Health (NCC-WCH) [15] guidelines scored the highest (both greater than 80%) and the New York State Department of Health (NY DoH) [20] and National Health Care for the Homeless Council (NHCHC) [21] guidelines scored the lowest (0% and 4%, respectively). Only seven CPGs [12], [13], [14], [15], [17], [22], [23] reportedly based their recommendations on an underlying systematic review.

Editorial independence was the domain with the lowest scores across guidelines (mean 26% of the maximum possible score, range 0–75%). CPGs infrequently described how the views of the funding body may or may not have influenced the content, and eight guidelines (33%) did not provide any information on conflicts of interest for the CPG developers. Of the 16 (66%) CPGs that did provide information on competing interests, only one guideline reported that they discussed and resolved their conflicts [23].

In the overall assessment, 13 guidelines (54%) were recommended, seven (29%) were recommended with modifications, and four (17%) were not recommended (Table 2). The four guidelines that were not recommended had little to no evidence base and lacked editorial independence. All other guidelines were recommended provided that they still needed improvement in one or more domains.

## Discussion

The overall quality of the 24 guidelines for glycemic control in DM2 was highly variable, and no guideline scored well in all domains of quality. There was also significant variability across domains within guidelines. Guidelines consistently scored well in the domain of clarity and presentation, suggesting that this component of guideline development may be easier to achieve or more highly valued by guideline development organizations. On the other hand, editorial independence was poorly addressed by almost all guidelines (the only exceptions were the CPGs developed by the American College of Physicians (ACP) [22], [23]). Perhaps guideline developers either do not appreciate the importance of conflict of interest disclosures and management, or choose not to address the issue in a transparent manner. There is considerable evidence that financial conflicts of interest are highly prevalent among CPGs in a variety of clinical areas [24], [25], [26], [27], and there is emerging evidence that conflict of interest may affect guideline recommendations [28].

Our assessment also suggests that guideline developers do not pay sufficient attention to the applicability of their recommendations to their target audiences and to implementation issues. Lack of attention to these issues has been noted in other studies examining the quality and usefulness of clinical practice guidelines [7], [9], [29].

Several studies have examined the quality of various cohorts of CPGs in diabetes, and findings vary. Bennett and colleagues [30] reported summary scores for the AGREE domain of rigor of development ranging between 17% and 100% across 11 CPGs from North America and the United Kingdom that examined oral agents for glycemic control. Eight of these guidelines had summary scores of less than 50%. Guidelines on the management of diabetes in pregnancy [31] also reported a great deal of variability in quality, with editorial independence the most problematic domain. Stone and colleagues [32] noted a great deal of variability across eight guidelines from Western Europe on the management of DM2, again with applicability and editorial independence scoring poorly. On the other hand, Mahmud and Mazza [33] scored all domains very high for five guidelines on preconception care in women with diabetes, with the exception of editorial independence. To our knowledge, no study has examined the broad spectrum of diabetes pharmacotherapy guidelines as in our study, which presents the largest cohort of published guidelines from around the globe.

Systematic reviews should form the basis for all high quality CPGs [1]. In our cohort of 24 guidelines, however, only seven (produced by five organizations) included or referenced an underlying systematic review. This suggests a fundamental problem with the majority of these CPGs. Even when present, the systematic reviews underpinning CPGs varied in quality, as indicated by the domain of rigor of development in AGREE II.

There are several important issues with regards to using AGREE II to appraise the quality of CPGs. First, the AGREE II domain of rigor of development does not encompass all important aspects of the quality of a systematic review, as does a quality assessment instrument developed specifically for that purpose, such as AMSTAR [34]. Second, and more importantly, AGREE II does not consider the relative importance of the six domains of quality: rigor of development is considered of equal importance to the other five domains. We think that this is problematic, and suggest that the domains of AGREE II should not be weighted equally. If the review underlying the guideline recommendations is either nonexistent or flawed (a low score on the domain of rigor of development), the guideline recommendations have a high risk of bias, and the other domains (no matter how well executed) are of little relevance in quality assessment.

The overall assessment in AGREE II of whether the CPG was recommended, recommended with modifications, or not recommended [11] is also problematic. There is no guidance in the AGREE II instrument as to how to make this assessment, and assessors may or may not weigh the various domains equally. For example, if most domains score high, but rigor of development scores low, an assessor might rate the CPG as “recommended”, and this could be misleading to potential users of the guideline. We suggest that AGREE II needs to be further revised to incorporate a hierarchy for appraisal, and to provide additional guidance on how to make the overall assessment.

This study has limitations, in addition to those imposed by AGREE II. Our cohort may not be representative of all diabetes guidelines, as we selected only those examining glycemic control for type 2 diabetes included in the NGC. Guidelines on other aspects of diabetes and those not in the NGC (which has minimum quality standards for inclusion) may differ in quality from those that we examined. In addition, the NGC does not contain all guidelines on diabetes: organizations choose to submit their guidelines to the NGC, and we did not search other sources for additional relevant guidelines.

We purposefully chose a low threshold for defining whether a systematic review was used to develop recommendations in the CPG. If we had imposed a more stringent definition such as one requiring a search of multiple bibliographic databases, assessment of quality of individual studies and of the body of evidence, and an explicit framework for developing recommendations from the body of evidence, the number of CPGs in our cohort that were considered to base recommendations on an underlying systematic review would have been far fewer.

In view of the potential impact of CPGs on health care delivery and patient outcomes, it is imperative that guidelines be of optimal quality. It is clear from this cohort of CPGs on glycemic control in DM2 that only a small minority of guidelines fulfill most criteria for a high quality guideline. The guideline user needs to beware, to critically appraise guidelines before use and to weigh the relative importance of the criteria for quality, starting with an assessment of whether a high quality systematic review underpins each recommendation.

## Supporting Information

Figure S1 **PRISMA flow diagram.** *From:* Moher D, Liberati A, Tetzlaff J, Altman DG, The PRISMA Group (2009). *P*referred *R*eporting *I*tems for *S*ystematic Reviews and *M*eta-*A*nalyses: The PRISMA Statement. PLoS Med 6(6): e1000097. doi:10.1371/journal.pmed1000097.
(TIF)
