# Supplementary figures and images for: Quality of Clinical Practice Guidelines for Glycemic Control in Type 2 Diabetes Mellitus
Source: PLoS One. 2013 Apr 5;8(4):e58625. doi: 10.1371/journal.pone.0058625 (PMC3618153; doi:10.1371/journal.pone.0058625)

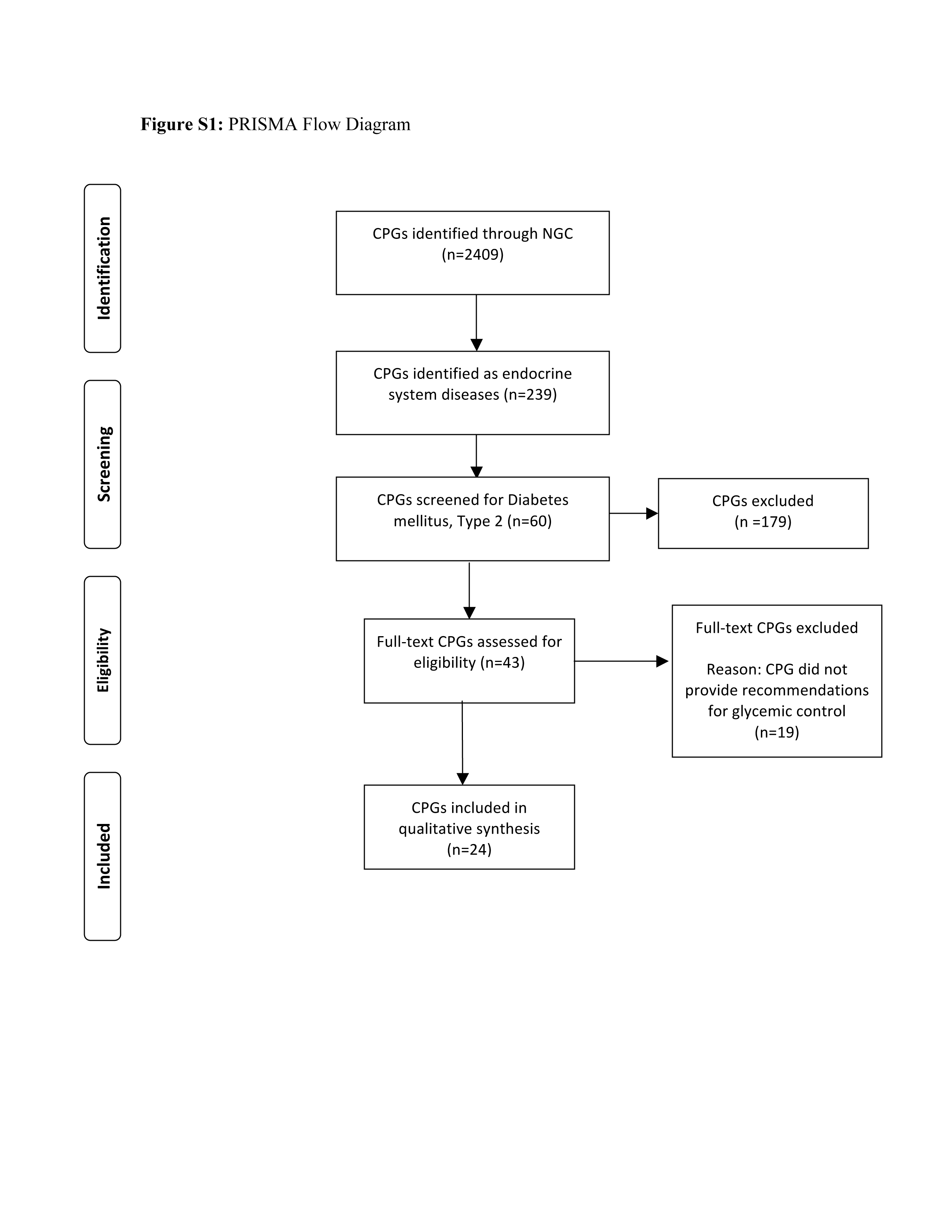

Supplement: Figure S1 — PRISMA flow diagram. From: Moher D, Liberati A, Tetzlaff J, Altman DG, The PRISMA Group (2009). Preferred Reporting Items for Systematic Reviews and Meta-Analyses: The PRISMA Statement. PLoS Med 6(6): e1000097. doi:10.1371/journal.pmed1000097. (TIF) [file pone.0058625.s001.tif]
